# Anti-Osteoporotic Effects of *Angelica sinensis* (Oliv.) Diels Extract on Ovariectomized Rats and Its Oral Toxicity in Rats

**DOI:** 10.3390/nu6104362

**Published:** 2014-10-16

**Authors:** Dong Wook Lim, Yun Tai Kim

**Affiliations:** 1 Food Resource Research Center, Korea Food Research Institute, Seongnam 463-746, Korea; E-Mail: neodw4015@kfri.re.kr; 2 Research Group of Food Functionality, Korea Food Research Institute, Seongnam 463-746, Korea; 3 Division of Food Biotechnology, Korea University of Science and Technology, Daejeon 305-350, Korea

**Keywords:** *Angelica sinensis* root, oral toxicity, osteoporosis, ovariectomized

## Abstract

*Angelica sinensis* root is one of the herbs most commonly used in China; it is also often included in dietary supplements for menopause in Europe and North America. In the present study, we examined the anti-osteoporotic effects of *A. sinensis* extract in an ovariectomized (OVX) rat model of osteoporosis as well as toxicity of the extract after repeated oral administration. The OVX rats were treated with 17β-estradiol (10 μg/kg i.p. once daily) or *A. sinensis* extract (30, 100, and 300 mg/kg, p.o. once daily) for four weeks. The bone (femur) mineral density (BMD) of rats treated with the extract (300 mg/kg) was significantly higher than that of the OVX-control, reaching BMD of the estradiol group. Markers of bone turnover in osteoporosis, serum alkaline phosphatase, collagen type I C-telopeptide and osteocalcin, were significantly decreased in the extract group. The body and uterus weight and serum estradiol concentration were not affected, and no treatment-related toxicity was observed during extract administration in rats. The results obtained indicate that *A. sinensis* extract can prevent the OVX-induced bone loss in rats via estrogen-independent mechanism.

## 1. Introduction

*Angelica sinensis* (Oliv.) Diels. (Chinese Angelica root; Danggui) has been used as a traditional Chinese Medicine (TCM) with a long history of use in China. It is still one of the herbs most commonly used in China, and as a dietary supplement in Europe and North America [1]. *A. sinensis* extract have also been reported to possess hepatoprotective [2], neuroprotective [3], anti-oxidant [4], anti-osteoarthritis [5], and anti-cancer [6] effects. The major active compounds of *A. sinensis* responsible for its diverse biological activities are phthalides, organic acids, polysaccharides, and flavones [7].

Pro-inflammatory cytokines such as interleukin-1 (IL-1), IL-6, and tumor necrosis factor-α (TNF-α) are well known regulators of bone metabolism. These cytokines are known as highly potent bone resorption cytokines [8,9,10,11] which can mediate increased bone turnover markers [12]. From the above reports it is hypothesized that the anti-inflammatory action of *A. sinensis* extract [13,14,15] might have potential anti-osteoporotic effects in an animal model via inhibition of bone turnover markers.

In the present study we examined the anti-osteoporotic effects of *A. sinensis* extract in an OVX-induced osteoporosis rat model and the toxicity of *A. sinensis* extract after repeated oral administration. Bone mineral density (BMD) of the femur was determined weekly using dual energy X-ray absorptiometry (DXA). Collagen type I C-telopeptide (CTx) and osteocalcin (OC) concentrations were assayed using ELISA. Serum estradiol concentration was determined by a radioimmunoassay (RIA). Automatic analyzer was used for serum biochemical determination of following parameters: aspartate aminotransferase (AST), alanine transaminase (ALT), gamma-glutamyltransferase (GGT), glucose (GLU), blood urea nitrogen (BUN), alkaline phosphatase (ALP), creatinine (CRE), and total protein (TP).

## 2. Experimental Section

### 2.1. Sample Preparation

The dried root of *Angelica sinensis* (Oliv.) Diels was purchased from Kapdang Co. (Seoul, Korea). The voucher specimen (#NP-1072) was deposited in the Functionality Evaluation Research Group, Korea Food Research Institute, Seongnam, Korea. *A. sinensis* (300 g) was extracts with 70% ethanol (3000 mL) for 3 h at 80 °C in a reflux apparatus. The process was repeated once, and the extract were combined and filtered through a membrane filter (0.45 µm; Millipore, Billerica, MA, USA). After removing the solvents via rotary evaporation, the remaining extract were vacuum dried (yield value 13.5% w/w). Z-ligustilide, as a major active ingredient of the extract, was purchased from ChromaDex (St. Santa Ana, CA, USA). The compositional analysis of Z-ligustilide from *A. sinensis* extract was performed using a high performance liquid chromatography (HPLC) system equipped with a Waters 1525 pump, a 2707 auto sampler and a 2998 PDA detector. The chromatographic separation was achieved at 30 °C on Waters Sunfire™ C18 (250 mm × 4 mm i.d., 5 μm particle size) column. The run time was set at 35 min; the flow rate was 1.0 mL/min; and the sample injection volume was 10 μL. The mobile phase was 0.1% (v/v) phosphoric acid (A)–100% acetonitrile (B) filtered through a 0.45 μm filter and degassed prior to use. Separation was achieved with gradient elution using 0.1% phosphoric acid as a solvent. The gradient was reduced by 90% from 0 to 10 min, 75% from 10 to 20 min and 50% from 20 to 30 min, and was increased by 90% from 30 to 35 min to equilibrate the column. The flow rate was set at 1.0 mL/min and the samples were detected at 307 nm.

### 2.2. Animal and Treatments

Female Sprague-Dawley (SD) rats, 12-weeks old, were purchased from Samtako, Gyeonggi-do, Korea. Animals were housed at two rats per cage in an air-conditioned room at 23 ± 1 °C, 55%–60% relative humidity, a 12 h light/dark cycle (07:00 lights on, 19:00 lights off), and were given a laboratory rodent diet 5010 (PMI Nutrition International, St. Louis, MO, USA). After acclimatization for one week, 13-week-old female SD rats were anesthetized with 2% of isoflurane and ovaries were removed bilaterally. A sham operation, during which the ovaries were just touched with forceps, was performed on the sham group. One week after surgery, rats were divided into five treatment groups: (1) sham + vehicle; (2) OVX + vehicle; (3) OVX + 17β-estradiol (E2, 10 μg/kg once daily, i.p); (4) OVX + *A. sinensis* extract 30 mg/kg; (5) OVX + *A. sinensis* extract 100 mg/kg; and (6) OVX + *A. sinensis* extract 300 mg/kg. *A. sinensis* extract was dissolved in distilled water administered orally in a volume of 5 mL/kg once daily. E2 was dissolved in distilled water with 1% dimethyl sulfoxide (DMSO) and 0.1% Tween 20. All groups were treated for four weeks. During the experimental period, body weight and femur bone mineral density (BMD) were determined weekly. At the end of the treatment period, the rats were fasted for 12 h, and blood was collected via the abdominal aorta. Uterus and other organs (heart, liver, spleen, and kidney) were dissected, washed with saline solution, and weighed for analysis. Uterus index (mg/g) was calculated by dividing the uterus by the body weight. All animal experiments were carried out according to the guidelines of the Korea Food Research Institutional Animal Care and Use Committee (KFRI-M-12024).

### 2.3. Rat Toxicity Studies

Repeat-dose oral toxicity study was carried out according to the Organization for Economic Cooperation and Development guideline 407. The 28 days oral dose study in SD rats was performed to assess the general toxicity of *A. sinensis* extract in rats (*n* = 5/sex/dose group) at doses of 1000 and 2000 mg/kg following daily oral administration. Groups 1 received 5 mL/kg body weight of distilled water and served as normal control. Groups 2 and 3 received *A. sinensis* extract at doses of 1000 and 2000 mg/kg body wt, respectively. *A. sinensis* extract was administered daily for 28 days the same time and observed at least twice daily for morbidity and mortality. Body weights of the animals were evaluated weekly. At the end of the treatment period, the rats were fasted for 12 h, and blood was collected via the abdominal aorta for biochemical analysis.

### 2.4. Bone Mineral Density Measurements

The BMD of femur was measured by a PIXImus (software version 1.42, GE Lunar PIXImus, GE Healthcare, WI, USA), dual energy X-ray absorptiometer (DXA) for bone density assessment in small laboratory animals. Calibration of the instrument was conducted as recommended by the manufacturer. Quality control with BMD (0.0553 g/cm^2^) and percentage fat composition (16.7%) of the phantom were also performed each time the instrument was switched on. The percent coefficient of variation for rat BMD at the femur was 1.5%–2.0%. All rats were placed in the same direction.

### 2.5. Serum Estradiol and Bone Marker Analysis

The serum samples were prepared by centrifugation of the collected blood samples (1000× *g* for 15 min at 4 °C) and stored at −80 °C for biochemical analysis. The serum concentrations of aspartate aminotransferase (AST), alanine transaminase (ALT), gamma-glutamyltransferase (GGT), glucose (GLU), blood urea nitrogen (BUN), alkaline phosphatase (ALP), creatinine (CRE), and total protein (TP) were determined using an automatic analyzer (ADVIA 1650, Bayer, Tokyo, Japan). Serum hormone level was determined by radioimmunoassay (RIA). The estradiol RIA was performed according to the instructions accompanying a Coat-a-Count kit (Diagnostic Products, Los Angeles, CA, USA). Serum concentrations of the bone formation marker, osteocalcin (OC) [16] were assayed using a rat ELISA kit (Metra OC, Quidel Corporation, San Diego, CA, USA). Serum levels of telopeptides of collagen type I (CTx), which correlate with bone resorption with high levels indicating increased osteoclastic activity [17], were analyzed using ELISA kits (Serum CrossLaps, Nordic Bioscience, Herlev, Denmark; Metra Serum Pyd, Quidel Corporation, San Diego, CA, USA).

### 2.6. Statistical Analysis

All data were presented as the mean ± standard deviation (SD). Statistical analysis was performed by one-way analysis of variance (ANOVA) with Tukey test to evaluate significant differences between groups using GraphPad Prism 5 (GraphPad Software Inc., La Jolla, CA, USA). *p* < 0.05 was considered statistically significant.

## 3. Results

### 3.1. Compositional Analysis of Z-Ligustilide from A. sinensis Extract

**Figure 1:**
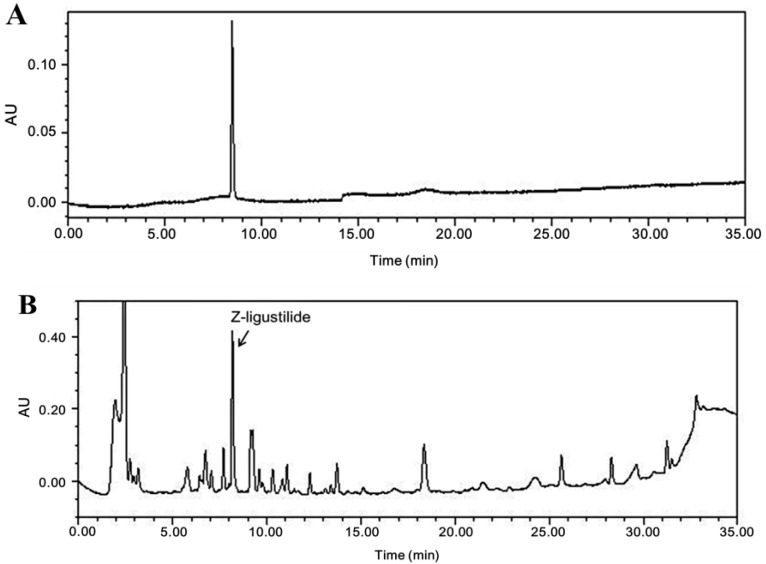
High performance liquid chromatography (HPLC) chromatograms of Z-ligustilide (**A**) and *A. sinensis* extract (**B**). Detection was performed using a photodiode array detector. *X*-axis is retention time (min); *Y*-axis is absorbance unit (AU).

*A. sinensis* extract was monitored at 307 nm for Z-ligustilide (Figure 1). *A. sinensis* extract was standardized to contain 1.9% ± 0.21% Z-ligustilide.

### 3.2. Body Weight Gain, Uterus Index, and Bone Mineral Density in Rats Treated with A. sinensis Extract

**Figure 2:**
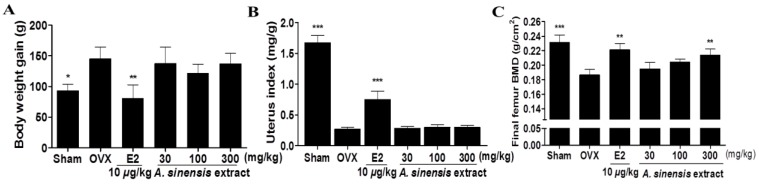
Effects of *A. sinensis* extract on body weight gain (**A**); uterus index (**B**); and bone mineral density (BMD) (**C**) in ovariectomized (OVX) rats. The body weight gain was calculated by the equation: final body weight—initial body weight. Uterus was dissected, washed with saline, and weighted. The BMD values were determined by dual energy X-ray absorptiometry. Here and in Figure 3, data are mean ± SD values (*n* = 10 per group). *** *p* < 0.001, ** *p* < 0.01, and * *p* < 0.05, as compared with the OVX control group.

After four weeks of treatments, the body weight gain of the E2 group was significantly lower than that of the OVXcontrol group. However, there was no significant difference in the body weight gain of *A. sinensis* extract-treated groups (Figure 2A). OVX caused atrophy of uterine tissue, indicating the success of the surgical procedure, and in the E2 group the uterus index increased significantly compared to the OVXcontrol group. However, *A. sinensis* extract-treated groups did not show an effect on the uterus index following OVX (Figure 2B). After four weeks of treatments, the final femur bone mineral density (BMD) of the *A. sinensis* extract 300 mg/kg-treated group was significantly higher than that of the OVXcontrol group (Figure 2C).

### 3.3. Serum Bone Marker in Rats Treated with A. sinensis Extract

**Figure 3:**
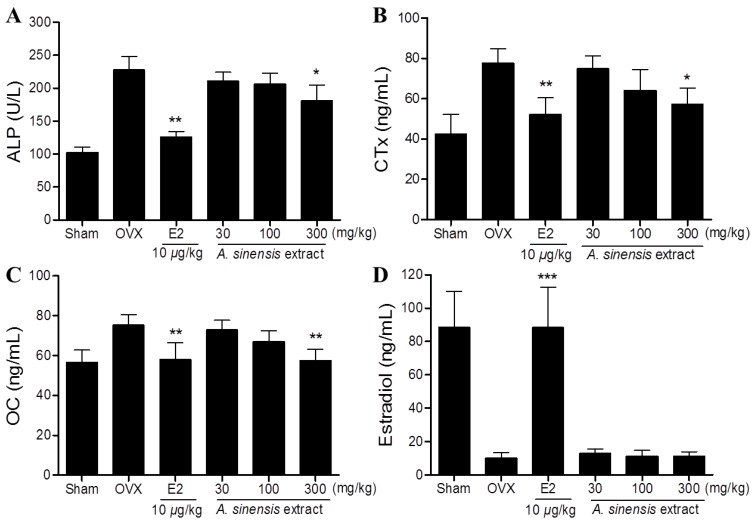
Effects of *A. sinensis* extract on serum alkaline phosphatase (ALP) (**A**); collagen type I C-telopeptide (CTx) (**B**); osteocalcin (OC) (**C**); and estradiol (**D**) concentrations in OVX rats. *** *p* < 0.001, ** *p* < 0.01, and * *p* < 0.05, as compared with the OVX control group.

Serum ALP, CTx, and OC concentrations in the OVX-control group were significantly higher compared to the sham group. After four weeks treatments, the *A. sinensis* extract 300 mg/kg-treated group showed significantly lower serum ALP, CTx, and OC concentrations compared to the OVX-control group (Figure 3). In case of serum estradiol, the *A. sinensis* extract treated groups were not significantly different from the OVX-control group (Figure 3D).

### 3.4. Effects of A. sinensis Extract on Sub-Chronic Toxicity

No significant differences in body weight were observed between normal and treated groups during the administration period (Table 1). Following sub-chronic toxicity test (28 days), no changes of organs weight and serum biochemical parameters were observed between the control and treatment groups (Table 2 and Table 3).

**Table 1:** Mean body weight of rats after 28 days treatment with *A. sinensis* extract.

Week	Mean Body Weight (g ± SD)					
	Male			Female		
	Normal	Extract (mg/kg)		Normal	Extract (mg/kg)	
		1000	2000		1000	2000
0	234.3 ± 9.1	227.6 ± 10.3	230.0 ± 8.5	187.6 ± 3.8	185.7 ± 4.9	182.8 ± 5.0
1	281.7 ± 13.5	273.8 ± 12.9	279.5 ± 10.4	199.0 ± 10.4	210.8 ± 7.1	212.4 ± 10.8
2	321.3 ± 19.2	312.9 ± 16.4	318.5 ± 12.6	219.0 ± 12.2	229.0 ± 9.8	230.1 ± 7.8
3	343.1 ± 22.8	338.9 ± 16.6	341.2 ± 15.5	225.6 ± 8.5	230.9 ± 9.1	228.4 ± 10.0
4	370.1 ± 20.9	366.2 ± 19.3	368.5 ± 15.8	236.4 ± 8.6	243.7 ± 10.5	240.6 ± 11.4

**Table 2:** Mean organ weight of rats after 28 days treatment with *A. sinensis* extract.

Organ	Mean Weight (g ± SD)					
	Male			Female		
	Normal	Extract (mg/kg)		Normal	Extract (mg/kg)	
		1000	2000		1000	2000
Heart	0.9 ± 0.0	0.9 ± 0.0	0.8 ± 0.1	0.7 ± 0.0	0.7 ± 0.0	0.6 ± 0.1
Liver	10.1 ± 1.0	9.9 ± 1.5	9.9 ± 1.0	6.6 ± 0.5	6.5 ± 0.7	6.6 ± 0.0
Spleen	0.7 ± 0.1	0.7 ± 0.1	0.6 ± 0.0	0.5 ± 0.1	0.5 ± 0.1	0.6 ± 0.0
Kidney	1.2 ± 0.1	1.2 ±0.1	1.2 ± 0.0	0.8 ± 0.0	0.7 ± 0.1	0.7 ± 0.0
Testis	1.9 ± 0.2	2.0 ± 0.1	2.0 ± 0.2	-	-	-
Uterus	-	-	-	0.8 ± 0.1	0.7 ± 0.0	0. 8 ± 0.0

**Table 3:** Biochemical parameters of rats after 28 days treatment with *A. sinensis* extract.

Serum Parameter	Mean Weight (g ± SD)					
	Male			Female		
	Normal	Extract (mg/kg)		Normal	Extract (mg/kg)	
		1000	2000		1000	2000
AST (U/L)	66.3 ± 12.2	76.0 ± 8.5	72.2 ± 10.5	64.3 ± 10.8	60.5 ± 6.4	62.8 ± 5.5
ALT (U/L)	29.8 ± 3.4	25.0 ± 1.41	26.0 ± 4.2	24.7 ± 4.7	26.0 ± 2.8	24.0 ± 2.5
GGT (U/L)	5.2 ± 3.7	4.0 ± 1.4	4.5 ± 2.4	4.2 ± 3.1	3.0 ± 0.0	3.8 ± 0.0
GLU (mmol/L)	6.3 ± 0.85	6.1.0 ± 0.50	5.9 ± 2.01	5.8 ± 1.08	6.0.0 ± 0.75	5.9 ± 0.67
BUN (mg/dL)	24.1 ± 2.5	25.7 ± 1.9	24.2 ± 3.4	19.6 ± 7.5	22.5 ± 3.7	22.4 ± 3.0
ALP (U/L)	582.0 ± 20.1	652.0 ± 9.9	630.0 ± 4.8	293.2 ± 52.5	312.5 ± 54.5	318.5 ± 50.2
CRE (mg/dL)	0.21 ± 0.03	0.15 ± 0.07	0.18 ± 0.01	0.21 ± 0.03	0.20 ± 0.00	0.20 ± 0.01
TP (g/dL)	6.0 ± 0.23	6.2 ± 0.64	6.2 ± 0.52	6.0 ± 0.25	5.9 ± 0.07	6.0 ± 0.05

AST, aspartate aminotransferase; ALT, alanine transaminase; GGT, gamma-glutamyltransferase; GLU, glucose; BUN, blood urea nitrogen; ALP, alkaline phosphatase; CRE, creatinine; TP, total protein.

## 4. Discussion

Our finding demonstrated that four weeks of treatment with *A. sinensis* extract significantly decreased the BMD loss in the femur and inhibited the bone turnover markers—serum ALP, OC, and CTx levels compared to the OVXcontrol group without influencing estrogen level.

Bone loss caused by estrogen deficiency in both experimental animals and humans is generally due to an increase in osteoclastic bone resorption [18]. OVX rats, which exhibit most of the characteristics of human postmenopausal osteoporosis [19], are widely used as a model for the evaluation of potential osteoporosis treatments [20].

In our experiments, OVX resulted in significant decrease in femur BMD after four weeks. The BMD loss was accompanied by a significant increase in bone remodeling, as evidenced by the increased biochemical bone turnover markers, such as serum ALP [21,22,23], CTx, and OC levels [24]. In the present study, oral administration of *A. sinensis* extract at the dose 300 mg/kg significantly decreased BMD loss, which was accompanied by the decrease in serum ALP, CTx, and OC levels compared to a OVXcontrol group. These results suggest that *A. sinensis* extract decreases bone loss by inhibiting bone turnover induced by OVX.

OVX dramatically increases body weight, while E2 treatment prevents body weight gain [25]. Estrogen deficiency induced body fat accumulation and subsequently caused an increase in body weight [26]. Heine *et al.* demonstrated that estrogen receptor (ER) knockout mice have higher fat mass and lower energy expenditure than wild-type mice [27]. Estrogen may be involved directly in energy metabolism by binding to ER within the abdominal and subcutaneous fat tissue [28]. Estrogen expresses its activities by binding to different ERs, ERα and ERβ. ERβ is more abundant than ERα in bone tissue, while ERα it is mainly distributed in reproductive cells and is the dominant receptor mediating the effects of E2 in breast and uterus [29]. In our experiments, oral administration of *A. sinensis* extract did not affect serum estradiol concentration, body weight gain, and uterotrophic activity in OVX rats. These results suggest that, the *A. sinensis* extract might have anti-osteoporotic effects in OVX rats, without the influence of hormones such as estrogen.

It is important to note that *A. sinensis* extract contains ferrulic acid, which is a potent antioxidant and a free radical scavenger [30]. It has been demonstrated that oxidation-derived free radicals increase bone resorption by promoting osteoclastic differentiation [31]. Ma *et al.* demonstrated that Z-ligustilide from the essential oil of *A. sinensis* inhibits the OVX-induced serum interleukin-1β (IL-1β) and tumor necrosis factor-α (TNF-α) levels [32]. IL-1β is known as a highly potent bone-resorptive cytokine [9] and TNF-α appears to synergize with IL-1 to increase bone resorption [11]. The effects of *A. sinensis* on bone thus appear to be related to its high contents of the ferulic acid or Z-ligustilide.

The toxicity of *A. sinensis* extract must be evaluated before it can be used in the drug or supplement development. In the rat toxicity studies, *A. sinensis* extract was administered orally at doses 1000 and 2000 mg/kg/day. *A. sinensis* extract caused no changes that could be considered toxicologically significant. Repeated oral administration of *A. sinensis* extract to rats for 4 weeks resulted in no toxicological changes in any of the clinical signs, body weight changes, serum biochemistry, necropsy findings, and relative organ weights. Thus, under the present experimental conditions, the No Observable Adverse Effect Level (NOAEL) of *A. sinensis* extract was assumed to be 2000 mg/kg/day for both male and female rats.

## 5. Conclusions

In conclusion, *A. sinensis* extract can prevent OVX-induced bone loss with efficacy comparable to that of estrogen. The findings obtained suggest that *A. sinensis* extract could be an effective natural alternative for the prevention of postmenopausal osteoporosis. Moreover, in the safety evaluation studies, *A. sinensis* extract was shown to be safe up to 2000 mg/kg/day for 4 weeks of administration in rats. The results presented here provide a foundation for clinical evaluation and demonstrate the potential of *A. sinensis* extract as an herbal drug.
